# Color Stability of Zinc Oxide Poly(methyl methacrylate) Nanocomposite—A New Biomaterial for Denture Bases

**DOI:** 10.3390/polym14224982

**Published:** 2022-11-17

**Authors:** Marcin Szerszeń, Mariusz Cierech, Jacek Wojnarowicz, Bartłomiej Górski, Elżbieta Mierzwińska-Nastalska

**Affiliations:** 1 Department of Prosthodontics, Medical University of Warsaw, 02-006 Warsaw, Poland; 2 Laboratory of Nanostructures, Institute of High Pressure Physics, Polish Academy of Sciences, 01-142 Warsaw, Poland; 3 Department of Periodontal and Oral Mucosa Diseases, Medical University of Warsaw, 02-097 Warsaw, Poland

**Keywords:** zinc oxide nanoparticles, poly(methyl methacrylate), color, chemical stability

## Abstract

(1) Background: The purpose of this in vitro study was to evaluate the color change and stability of a zinc oxide nanoparticle–poly(methyl methacrylate) (ZnO NP–PMMA) nanocomposite for denture base material after immersion in different dietary and cleaning agent solutions. (2) Methods: One hundred samples were prepared and divided into four equinumerous groups depending on the weight content of ZnO NPs. The color coordinates (CIE L*a*b*) were measured using a digital colorimeter, ColorReader (Datacolor AG Europe, Rotkreuz, Switzerland), before and after immersion of the specimens in five different solutions (distilled water, coffee, red wine, black tea, denture cleaning tablet solution) for 6 months. The color changes (ΔE) were calculated using Euclidean distance and analyzed by the Shapiro–Wilk test and the ANOVA/Kruskal–Wallis multiple comparison and adequate post hoc tests. (3) Results: All tested materials showed significant color changes after their exposure to all solutions. Color changes were greatest in the case of red wine and progressed with the duration of the study. (4) Conclusions: The modification of PMMA with ZnO nanoparticles is acceptable in aesthetic terms in 2.5% and 5% weight content; however, color changes are more noticeable with higher nanoparticle content and must be discussed with the patient prior to possible use.

## 1. Introduction

Poly(methyl methacrylate) (PMMA) resins are clinically used in prosthodontics in full and partial denture production for decades [1,2,3,4]. Despite the fact that there are many advantages of these materials, such as ease of laboratory processing, polishability, appropriate physicochemical properties, and no smell or taste after polymerization, the issues of microporosity and thus water absorption and susceptibility to microbial growth are still challenges in considering this material as ideal for removable prosthetic devices [5,6,7,8]. The appropriate composition of PMMA intended for the fabrication of dentures allows for satisfactory aesthetic effects not only in terms of artificial dentition, but also the so-called pink aesthetics, i.e., the color of the denture plate imitating atrophied soft tissues in the oral cavity and designed to distribute the chewing forces over a larger surface of the toothless prosthetic foundation [9]. 

PMMA can be modified with both organic and inorganic substances to improve its mechanical, tribological, aesthetic, or microbiological properties. Amid the development of nanotechnology, substances are increasingly doped on the nanoscale in order to change specific properties of the material [10,11]. The most desirable feature of such a modification is microbiological activity, which would reduce the possibility of the bacterial and fungal biofilm accumulation on the prosthesis base plate. The use of silver nanoparticles with proven antibacterial and antifungal activity is the most well-known modification of prosthodontic materials nowadays [12,13,14,15]. However, the dark brown color that results from the preparation of PMMA–nanosilver composite disqualifies it in terms of aesthetics, limits its applicability, and eliminates its relevance in routine clinical practice. The addition of nanosilver in composite materials may also affect the stability of the biomaterial in the oral cavity by increasing the release of metal ions with all their positive and negative effects [16].

Another well-known modification of acrylic material is the incorporation of nano-titanium particles aimed at improving both mechanical and microbiological properties. In the case of this modification, a whitish color of the nanocomposite was demonstrated, significantly limiting the use of dentures for repair or relining, where the new material is located only in the unsightly zone [17,18]. 

Regarding the chemical composition of these materials, the mechanical and functional properties including color change in the resin–matrix materials depends on the organic matrix and inorganic particles, and the type of polymerization initiator system [19,20,21,22]. A large filler content reduces the organic matrix’s content. Insufficient polymerization, water absorption, or the adsorption of water-soluble colored beverages such as coffee, red wine, etc., can all cause color changes. The type and degree of monomer conversion that establishes the required physicochemical characteristics influence the susceptibility of the organic matrix of the resin–matrix composite to retain coloration [23]. The authors previously reported the production of PMMA modified with zinc oxide nanoparticles and characterized its mechanical, microbiological, and cytotoxic properties [24,25]. Color and its durability when exposed to the coloring agents that are present in the oral cavity environment during everyday usage of dentures will significantly determine the prospective applicability of the aforementioned modifications in dental clinics [26]. 

Color stability can be assessed using either visual or instrumental techniques. The color change can be measured clinically or with special instruments that remove the subjective disturbance that occurs with visual color perception. It can be assumed that a standard observer notices a color difference as follows [27,28]:

- 0 < ΔE < 1—does not notice the difference;
- 1 < ΔE < 2—only an experienced observer notices the difference;
- 2 < ΔE < 3.5—an inexperienced observer also notices the difference;
- 3.5 < ΔE < 5—notices a clear color difference; and
- ΔE > 5—the observer has the impression of two different colors.

The assessment of color changes can also be analyzed according to the formula proposed by the National Bureau of Standards: NBS = ΔE × 0.92. The range of NBS units is as follows:

- 0.0–0.5, trace;
- 0.5–1.5, slight;
- 1.5–3.0, noticeable;
- 3.0–6.0, appreciable;
- 6.0–12.0, large (much); and
- >12.0, very much.

Colorimeters and spectrophotometers are common equipment for detecting color changes in restorative materials while minimizing subjective interference, and they enable the comparison of two colors within the same color space in the form of the ΔE parameter [29,30,31]. The light range of the illumination, the wavelength reflected or transmitted by the object, and the observation characteristics of the human observer can all alter objective evaluation of color parameters. For this reason, it is necessary to perform not only qualitative but also quantitative assessments and comparisons of the color characteristics of the newly created nanomaterial composites with those used on a daily basis in clinical work.

The aim of this study was to evaluate the manner in which the incorporation of zinc oxide changes the color properties of PMMA and the permanence of the obtained color after immersion in different dietary and therapeutic solutions. The first null hypothesis was that there is no significant difference in color changes between PMMA modified with ZnO nanoparticles and unmodified PMMA. The second null hypothesis was that there are no differences in color permanence of modified and unmodified PMMA, depending on the external solution environment.

## 2. Materials and Methods

### 2.1. Characteristics of ZnO Nanoparticles 

In this study, the author’s procedure [32] for microwave solvothermal synthesis (MSS) [33] was used to produce zinc oxide nanoparticles (ZnO NPs). Zinc acetate dihydrate (Zn(CH_3_COO)_2_·2H_2_O, pure for analysis, Chempur, Piekary Śląskie, Poland) and ethylene glycol (C_2_H_4_(OH)_2_, pure for analysis, Chempur, Piekary Śląskie, Poland) were used in the production protocol. Zinc oxide was obtained by dissolving zinc acetate in ethylene glycol. The reaction solution was placed into a covered Teflon vessel and heated using microwave radiation after 45 min of additional stirring. The microwave reactor MSS2 (IHPP PAS (Warsaw, Poland), ITeE-PIB (Radom, Poland), ERTEC (Wrocław, Poland)) was then set to 2.45 GHz with a power density of approximately 10 W/mL [34]. The reactions took 12 min to complete at a constant pressure of 3 bar and a microwave power of 3 kW. The resulting powder was sedimented, rinsed three times with deionized water (1 class, HLP 20 UV, Hydrolab, Straszyn, Poland), centrifuged (MPW-350, MPW Med Instruments, Warsaw, Poland), and dried in a freeze dryer (Lyovac GT 2, SRK Systemtechnik GmbH, Riedstadt, Germany) after the synthesis. The average particle size of the ZnO NPs used in this study was ≈30 nm, with a density of 5.24 g/cm^3^, a specific surface area of 39 m^2^/g, and phase purity. The scanning electron microscopy (SEM) and transmission electron microscope (TEM) examinations revealed great uniformity of nanoparticles in terms of size and shape. The characterization of the ZnO samples reported was carried out in a certified research laboratory with the accreditation number AB 1503 [35], which follows the PN-EN ISO/IEC 17025:2018-02 standard.

### 2.2. Preparation of Specimens 

The test material used in this study was thermally polymerized acrylic resin (Superacryl Plus, Spofa Dental, Jicin, Czech Republic). The mixing procedure involves a 3:1 powder–liquid volume ratio, which corresponds to 22 g of polymer and 10 mL of liquid monomer. A calculated amount of nanopowder was suspended in a liquid acrylic resin monomer and mechanically mixed for 60 s using a metal spatula. The estimated amount of PMMA was then added to achieve final weight concentrations of 2.5%, 5%, and 7.5%. Table 1 shows the precise weight composition of the constituent components. Modeling wax (Vertex Regular, Vertex-Dental BV, Centurionbaan, The Netherlands) was used to create 13 mm × 13 mm × 2 mm samples, which were then processed into acrylic samples using Class III hard plaster (Stodent, Zhermack, Badia Polesine, Italy) according to the standard flasking procedure. The wax samples were covered with a 0.025 mm thick polyethylene sheet (Divosheet, Vertex-Dental BV, Centurionbaan, The Netherlands) to obtain a flat surface and make the possible roughness of the material independent from machining and polishing. Afterwards, the material was subjected to traditional thermal polymerization in a polymerizer (PS-2, PEM, Warsaw, Poland), as recommended by the manufacturer (gradual temperature increase to 97 °C; polymerization period at 97 °C: 30 min). The control group consisted of acrylic specimens without nanoparticles. 

### 2.3. Color Measurement 

One hundred samples were created and divided into four equinumerous groups based on the weight content of ZnO NPs in order to compare the color of PMMA with the PMMA–ZnO nanocomposite. A colorimetric test was performed on the samples using a digital colorimeter (ColorReader, Datacolor AG Europe, Rotkreuz, Switzerland) with a wireless Bluetooth (BT) interface allowing connection to dedicated software. The data from the device were sent to the software, and then encoded in the CIE L*a*b* color space. The recording device’s specifications correspond to CIE (Commission internationale de l’éclairage—International Commission on Illumination) colorimetric test standards, which include a 10° observation angle and a D65 illuminant, with a built-in light source in the form of six light-emitting diodes, ensuring that the technical requirements are met. Measurements were made by one operator (M.S.) in the same room and under the same lighting conditions (dark room) and the samples were placed on the same test bench. The colorimeter was calibrated as recommended by the manufacturer with the included white standard before each series of data collection. 

CIE L*a*b* color space is a three-dimensional measurement system, where L* represents the clarity of an object ranging from black (0) to white (100), a* represents a measurement for the quality of red (a > 0) or green (a < 0), and b* represents a measurement for the quality of yellow (b > 0) or blue (b < 0). Each sample was subjected to five independent colorimetric measurements in various areas and on both sides of the prepared samples. The values obtained in this manner were compiled with the use of descriptive statistics (mean, SD), and then differences in the obtained colors between groups were calculated by means of the ΔE parameter according to the distance Formula (1). The ΔE value is the Euclidean distance between two colors in the color space, assuming that both colors have been described in the same space, and it is expressed as a number.

(1) $$\Delta E_{\mathrm{CIE}\;\mathrm{Lab}\;}=\sqrt{{\left(\Delta L\right)}^{2}+{\left(\Delta a\right)}^{2}+{\left(\Delta b\right)}^{2}},$$

In order to analyze color permanence depending on the external environment, the material prepared while comparing the color of unmodified and modified PMMA was used. Samples were divided first in terms of weight percentage content of ZnO NPs in the PMMA matrix, and subsequently into five groups depending on the sample immersion environment. Four solutions were applied: coffee (CO), red wine (RW), denture cleaning tablets (CT), and black tea (BT), which, when used by patients, could potentially change the color of the denture base plate. The control group contained samples immersed in distilled water (DW). The division by ZnO NP content, groups, and solutions used is illustrated in Table 2. The samples were fully immersed in the prepared solutions in separate polystyrene molds in order to prevent samples from contacting each other and stored without light access at the temperature of 23 °C ± 1 °C. Color measurements were performed before the immersion and subsequently after 1, 2, 3, 4, 5, 6, 7, 14, 28, 56, and 182 days. Each series of measurements was preceded with removing the samples from the vessels, rinsing them with distilled water, and drying them with dust-free cellulose towels. The solutions in which the samples were immersed were replaced every 24 h. The discrepancies in the obtained colors are presented in the form of the ΔE parameter. The unit ΔL, Δa, and Δb values were calculated for each sample as the difference between the value obtained before the immersion and after a given time of sample immersion in the solution. 

### 2.4. Statistical Analysis

The statistical analysis of obtained data was performed with Statistica 13 software (ver. 13.3, Tibco Software Inc., Palo Alto, CA, USA). Descriptive statistics including means and standard deviations were performed. The normal distribution of data was verified using Shapiro–Wilk tests. ANOVA or Kruskal–Wallis tests and then post hoc tests (Tamhane’s or Conover–Iman) were performed in groups with statistically significant differences. The level of significance for tests was set at *p* < 0.05.

**Table 2:** Mean ± standard deviation of ∆E parameter in relation to immersion solution and time of immersion with division into groups.

Group	Immersion Solution	∆E Parameter Value in Consecutive Days of the Study (Mean ± Standard Deviation)										
		1 d	2 d	3 d	4 d	5 d	6 d	7 d	14 d	28 d	56 d	182 d
A PMMA (Control group)	DW ^D,E^ CO ^B^ RW ^I^ BT ^2,B,C^ CT ^F,G^	1.69 ± 0.58 0.90 ± 0.14 2.63 ± 0.50 0.64 ± 0.29 1.07 ± 0.42	1.66 ± 0.70 0.97 ± 0.26 2.80 ± 0.74 1.12 ± 0.61 2.04 ± 0.55	1.36 ± 0.39 0.76 ± 0.55 3.56 ± 0.36 0.91 ± 0.57 1.88 ± 0.43	1.73 ± 0.86 1.17 ± 0.44 4.47 ± 0.82 1.71 ± 0.65 1.97 ± 0.65	1.54 ± 0.75 0.80 ± 0.22 6.43 ± 0.78 1.24 ± 0.49 1.94 ± 0.63	1.92 ± 1.30 0.94 ± 0.31 8.20 ± 0.62 1.39 ± 0.79 1.60 ± 0.56	1.93 ± 1.06 0.95 ± 0.41 10.08 ± 0.67 1.15 ± 0.78 1.61 ± 0.22	2.23 ± 0.36 1.18 ± 0.50 15.54 ± 0.33 1.18 ± 0.60 1.68 ± 0.37	2.14 ± 0.41 0.84 ± 0.36 20.70 ± 1.65 0.93 ± 0.29 2.81 ± 0.41	2.49 ± 0.67 1.21 ± 0.59 20.12 ± 2.38 1.70 ± 0.91 6.03 ± 1.10	3.09 ± 0.81 3.59 ± 0.34 28.66 ± 0.47 4.16 ± 0.61 5.85 ± 0.70
B PMMA-ZnONPs-2.5%	DW ^A^ CO ^1,4,C,D^ RW ^3,I^ BT ^A^ CT ^1,5,E,F^	0.44 ± 0.09 0.71 ± 016 2.05 ± 0.36 0.85 ± 0.10 0.76 ± 0.14	0.71 ± 0.26 1.38 ± 0.32 3.08 ± 0.41 0.62 ± 0.31 1.20 ± 0.09	0.63 ± 0.23 1.36 ± 0.26 4.32 ± 0.33 0.56 ± 0.24 1.41 ± 0.24	0.70 ± 0.15 1.45 ± 0.30 5.40 ± 0.50 0.61 ± 0.35 1.78 ± 0.23	0.57 ± 0.17 1.52 ± 0.07 6.64 ± 0.46 0.61 ± 0.14 1.58 ± 0.19	0.51 ± 0.15 1.32 ± 0.20 8.07 ± 0.27 0.57 ± 0.07 1.39 ± 0.12	0.68 ± 0.23 1.74 ± 0.16 9.89 ± 0.62 0.85 ± 0.41 3.12 ± 1.68	0.70 ± 0.26 2.21 ± 0.19 14.19 ± 0.44 0.62 ± 0.23 2.94 ± 0.43	0.90 ± 0.26 2.33 ± 0.25 20.39 ± 3.28 0.78 ± 0.35 5.52 ± 1.69	0.93 ± 0.12 3.39 ± 0.19 23.69 ± 3.99 0.72 ± 0.30 3.84 ± 0.61	2.66 ± 0.21 2.74 ± 0.26 24.65 ± 0.35 2.31 ± 0.22 5.52 ± 0.39
C PMMA-ZnONPs-5%	DW ^H^ CO ^1,D,E^ RW ^J,K^ BT ^A^ CT ^5,G,H^	3.59 ± 0.56 0.64 ± 0.22 2.90 ± 0.19 0.42 ± 0.11 1.78 ± 0.46	3.59 ± 0.58 1.13 ± 0.20 5.53 ± 0.31 0.39 ± 0.25 1.95 ± 0.23	3.66 ± 0.54 1.17 ± 0.25 6.71 ± 0.41 0.43 ± 0.23 1.62 ± 0.33	3.64 ± 0.51 1.72 ± 0.36 6.40 ± 0.23 0.56 ± 0.25 1.82 ± 0.45	3.60 ± 0.54 1.40 ± 0.60 9.08 ± 0.15 0.35 ± 0.09 1.57 ± 0.57	3.44 ± 0.52 1.88 ± 0.60 11.84 ± 0.59 0.66 ± 0.17 1.58 ± 0.80	3.57 ± 0.58 2.65 ± 1.51 12.91 ± 0.28 0.53 ± 0.17 2.39 ± 0.41	3.56 ± 0.63 3.06 ± 0.40 18.78 ± 1.53 0.45 ± 0.22 3.28 ± 1.33	3.82 ± 0.51 3.32 ± 0.29 25.66 ± 3.93 0.92 ± 0.51 5.99 ± 0.68	3.97 ± 0.58 2.84 ± 0.48 30.80 ± 6.25 0.75 ± 0.44 5.40 ± 0.36	2.96 ± 0.29 2.98 ± 0.24 27.13 ± 0.43 1.77 ± 0.42 9.89 ± 1.19
D PMMA-ZnONPs-7.5%	DW ^2,4,B^ CO ^3,I,J^ RW ^K^ BT ^2,4,B^ CT ^3,I^	0.79 ± 0.21 4.20 ± 0.88 6.23 ± 1.77 0.98 ± 0.59 3.29 ± 0.84	0.99 ± 0.55 5.71 ± 0.86 8.00 ± 1.52 1.09 ± 0.23 5.39 ± 0.14	0.98 ± 0.37 6.93 ± 0.71 9.25 ± 2.14 1.16 ± 0.38 5.79 ± 0.51	1.36 ± 0.19 7.97 ± 1.03 10.40 ± 1.66 0.82 ± 0.22 6.42 ± 0.39	1.20 ± 0.15 7.52 ± 1.08 12.23 ± 1.48 0.76 ± 0.35 6.32 ± 0.20	1.49 ± 0.27 7.86 ± 0.91 14.29 ± 1.59 0.80 ± 0.10 6.64 ± 0.34	1.44 ± 0.23 8.53 ± 1.29 15.93 ± 1.43 0.95 ± 0.33 7.00 ± 0.14	1.36 ± 0.28 8.83 ± 1.13 22.29 ± 1.51 0.90 ± 0.18 7.70 ± 0.66	2.11 ± 0.24 9.19 ± 0.99 31.73 ± 3.45 1.44 ± 0.39 6.45 ± 1.53	1.85 ± 0.44 12.30 ± 1.00 30.53 ± 1.59 1.24 ± 0.23 7.15 ± 0.96	1.74 ± 0.31 4.13 ± 0.89 31.04 ± 1.78 1.31 ± 0.16 7.50 ± 0.17
	Mean ∆E	1.83	2.47	2.72	3.11	3.34	3.82	4.40	5.58	7.35	8.00	8.63

^1^ Statistically insignificant difference in post hoc Conover–Iman with comparison to ADW, ^2^ statistically insignificant difference in post hoc Conover–Iman with comparison to ACO, ^3^ statistically insignificant difference in post hoc Conover–Iman with comparison to ARW, ^4^ statistically insignificant difference in post hoc Conover–Iman with comparison to ABT, ^5^ statistically insignificant difference in post hoc Conover–Iman with comparison to ACT, ^A–K^ the same letter means that the groups were homogenous in Conover–Iman post hoc comparison.

## 3. Results

### 3.1. Comparison of Color of Modified and Unmodified PMMA

**Figure 1:**
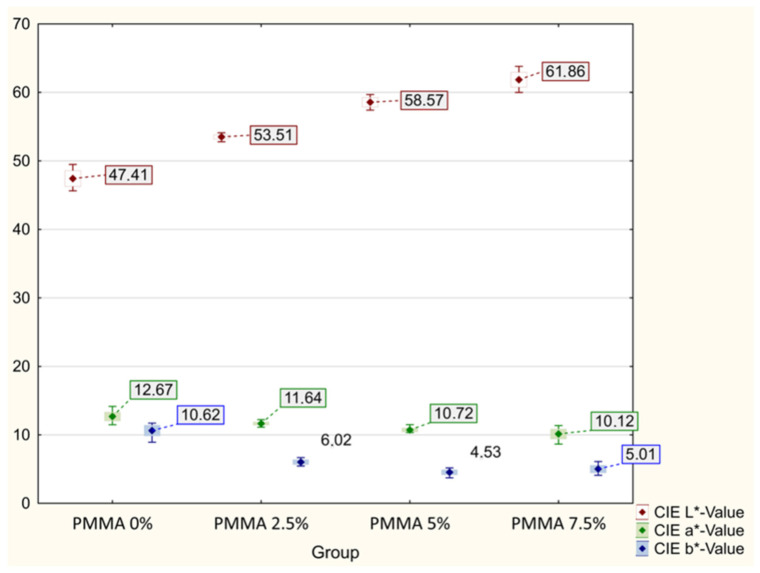
Comparison of mean values in CIE L*a*b* color space of modified and unmodified PMMA.

All tested samples in this part of the study, regardless of the group, showed the highest recorded mean values for the CIE L* component (lightness), and they increased with an increase in the weight content of ZnO nanoparticles in the range of 47.41 for the control group (PMMA) to 61.86 for the PMMA-ZnONPs-7.5% group. The differences in the L* value range were statistically significant between each group (ANOVA *p* < 0.000001; Tamhane’s post hoc *p* < 0.000001). The mean results of the CIE a* (green ↔ red) component decreased with the weight content of nanoparticles, reaching 12.67 for PMMA and 10.12 for PMMA with the highest content of Zn ONPs. The CIE b* (blue ↔ yellow) component also varied depending on the nanoparticle content in the range of 10.62 for PMMA to 5.01 for 7.5% Zn ONP content. The differences for the CIE a* and b* components were statistically significant for the individual groups (ANOVA *p* < 0.000001) in addition to the post hoc comparison between the PMMA-ZnONPs-5% and PMMA-ZnONPS-7.5% groups. The calculated ∆E showed differences in the range of 7.705–15.708 compared to the control group. The NBS parameter allowing for the qualitative presentation of the ∆E discrepancy results showed that the color of the samples in individual groups differed noticeably from high to very high. The mean values with standard deviations, ∆E in relation to control group, NBS, statistical data of PMMA samples, and digital representation of mean color of materials with different ZnO NP content are shown in Table 1 and Figure 1.

### 3.2. Color Permanence of Modified and Unmodified PMMA, Depending on the External Environment

**Figure 2:**
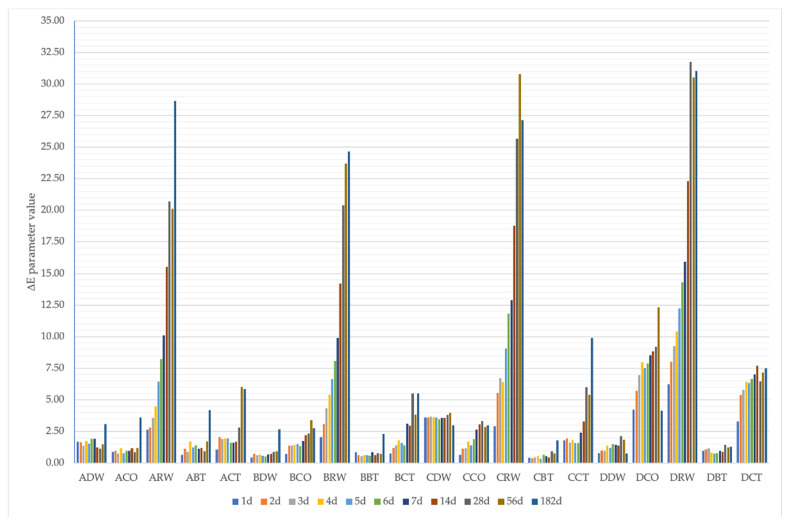
Comparison of mean values of ∆E parameter according to subgroup (immersion solution) and time.

All the tested samples showed color changes from the first day of the experiment, which progressed with the duration of the tests. The greatest color differences were noted for samples stored in red wine, reaching a ∆E value of over 31. The material placed in a black tea solution was characterized by the highest color stability. Regardless of the dye medium used, the greater the color changes, the greater the weight content of zinc oxide nanoparticles in the nanocomposite. Considering only the duration of the test, the minimum color change was noted on the 1st day of the research for samples in group B (2.5 wt % ZnO NP–PMMA) and was ∆E 0.96. The maximum color change for this criterion was seen for the material with 7.5 wt % ZnO NP–PMMA (group D) on day 56 of the study (∆E 10.61), but the difference in this case between day 56 and day 182 of the study was not statistically significant. The test time significantly influenced the changes in color regardless of the medium in which the samples were soaked—on the first day, the mean change for all samples was ∆E 1.83 and progressed until the change of ∆E 8.63 for the last day of the test. It is worth noting that the average values of ∆E changes progressed much faster for the samples with higher content of ZnO NPs in the nanocomposite, while the increase in changes stabilized over time, unlike pure PMMA samples, whose color changed gradually throughout the test. The exact results of the differences in mean ∆E values, along with statistically significant differences in the groups and between them, are presented in Table 2. A graphical representation of changes in the delta E parameter with a breakdown into groups and days of measurements is shown in Figure 2. 

## 4. Discussion

The main goal of dental prosthetics is the reconstruction of lost tissues within the stomatognathic system. In order for the restorations to be fully accepted by the patient, they must look natural and be imperceptible to the environment. The specificity of materials used in the oral cavity must therefore not only meet the strength and biocompatibility standards for the host tissues, but also the increasingly restrictive biomimetic standards for imitating the tissues of the tooth or mucosa. We were the first to create a ZnO–PMMA nanocomposite to reduce the accumulation of microorganisms on the material’s surface [36]. Satisfactory microbiological properties have been demonstrated with acceptable mechanical properties and low cytotoxicity [24,25]. Before introducing the above modifications to a wide range of use, it is necessary to evaluate the aesthetics of the material, including its color and color stability. 

Both null hypotheses were disproved due to the existing statistical differences between the colors of the samples of PMMA and those modified with ZnO nanoparticles, and the color changed statistically significantly, regardless of the coloring agent/external environment of the substance used in the study. The research showed that the CIE L* component increases with the increase in the zinc oxide content in individual nanocomposites, which proves that the material is whitened. Each test group showed a color change defined by the standard observer as two different colors (ΔE > 5). In the NBS classification, this change is defined from large to very large. The value of the L component for the 2.5%, 5%, and 7.5% nanocomposites increased by 13%, 22%, and 30%, respectively, compared to the control group. The whitening of the material after ZnO incorporation was also noticed by Rudolf et al. for a 2% PMMA–ZnO nanocomposite as well as by Cierech et al. in preliminary studies preceding this publication [37,38]. Smaller differences were observed in the CIE a* component, where the red intensity decreased, and for the 7.5% nanocomposite, the decrease was 20%. In the CIE b* component for the 7.5% nanocomposite, the largest decrease in value compared to the control group was observed, which amounted to as much as 47%. The results are consistent with the work of Kamonkhantikul et al., where the color change of PMMA modified with silanized and non-silanized ZnO was investigated, creating 1.25%, 2.5%, and 5% composites [39]. The ΔE for the 5% nanocomposite was 19–22, which gives the impression of two different colors. For the 5% silanized composite, an increase in the CIE L* parameter by 26% and a decrease in the CIE a* and CIE b* parameters by 23% and 65%, respectively, were observed. In the subjective opinion of the authors, as dentists with clinical experience in dental prosthetics, the 2.5% and 5% nanocomposites are aesthetically acceptable and could be used in the fabrication of removable denture plates. Before potential clinical use, however, a color key should be prepared to obtain patient approval of the expected shade of the denture plate. The problem of color change after ZnO incorporation can also be solved by adding an appropriate pigment, which, by changing the color of the material, would make the prosthesis even more similar to the shade of the patient’s mucosa. The authors are not aware of any previous publications dealing with this issue. 

Acrylic resin is considered the least stable in terms of color compared to other materials used in the production of long-term restorations, such as composites or ceramics [40]. Even though the prosthesis plate is constantly exposed to the coloring agents present in food and cleaning agents, with proper hygiene and compliance with medical recommendations, it is possible to maintain the obtained aesthetic effect for many years. In this study, premeditated specimens were covered with a polyethylene sheet prior to wax conversion to standardize and make specimen roughness independent of subsequent processing and polishing steps. In reality, however, properly carried out and repeated activities aimed at adequately reducing the roughness of prosthetic restorations seem to be one of the most important measures to prevent staining, external discoloration, and the deposition of denture plaque. In addition, as the hygienic activities performed by patients, such as brushing dentures, or modifications of dentures by dental professionals also affect the formation of micro or macro surface roughness, the assessment of the need to polish the prosthetic restoration should be one of the obligatory stages during follow-up visits.

The study used various coloring media that the denture plate may most often come into contact with. The duration of the study was arbitrarily set at 182 days. Unfortunately, there are no studies that would directly show the impact of laboratory PMMA discoloration time on the clinical application scenario. The range of factors influencing possible discoloration related to individual food preferences requires the conclusion that such indicators would be difficult to define; we consider this to be one of the limitations of the study. From our searches in the available literature, similar studies on color changes differed significantly in terms of the staining period and ranged from 7 to 180 days [41,42,43,44]. In our study, we wanted to show the trend between the duration of the coloring agent and the actual color change of ZnO NP–PMMA. Considering that materials with an admixture of nanoparticles are relatively new solutions, and their physico-chemical and aesthetic properties are still the subject of many studies, we wanted our study to also decide whether to accept or at least indicate the limitations of these materials in the aesthetic context. General knowledge about removable prosthetic restorations and the limitations resulting from the characteristics of classic polymethyl methacrylate indicate that full dentures should be replaced after a period of about 5 years of use, but the material we are testing could also potentially be used as an element of cast metal partial dentures or even long-term full-arch reconstructions based on intraosseous implants, the service life of which is significantly extended compared to classic plate removable restorations [45,46,47]. 

It was found that the dynamics of the color change of composites decreased significantly after 4 weeks of observation. After this period, the color was basically saturated, and the color changes of the materials were negligible. The 7.5% nanocomposite discolored the fastest, achieving results similar to its final coloring on the 7th day, which is why the 7th day of the test was considered the most appropriate for comparing the color stability of composites. 

It was observed in the study that distilled water and black tea as coloring media had the least effect on the color change of materials. For 7.5% nanocomposites on the 7th day of the study, the color changes were 1.44 ± 0.23 and 0.95 ± 0.33, respectively, which correspond to a slight change in the NBS classification. Red wine was the strongest staining medium, as it was the only one on the 28th day of observation to obtain a ΔE result above 20 both for the control group and individual study groups. Compared to pure PMMA, the lower concentrations of nanocomposites (2.5% and 5%) behaved similarly or underwent slightly more discoloration. The 7.5% nanocomposite behaved completely differently, and turned out to be much less color-stable than the other nanocomposites. This tendency was most visible for highly colored media such as CO, RW, and CT. The ranges of ΔE CO for the 2.5% and 5% nanocomposites were 1.74–2.65 and 8.53 for the 7.5% nanocomposite. For RW, the range was 9.89–12.91 compared to 15.93 and for CT, it was 3.12–2.39 compared to 7.00.

The explanation of such results may be the fact that the PMMA matrix is able to accept and stably integrate into its structural network a certain amount of ZnO nanoparticles. This was proved in studies of ZnO NPs’ release into the environment, where a 7.5% nanocomposite after 7 days of incubation showed release at the level of 3.5 mg/mL, while the release from 2.5% and 5% nanocomposites was at a similar level and amounted to 2.2 mg/mL and 2.1 mg/mL, respectively [25]. The increase in release by 62% shows that a large amount of nanoparticles are unstable in the polymer matrix and go to the external environment. Thus, the voids created in this mechanism can incorporate pigments from the environment and thus be responsible for a significant color change. 

Another possible mechanism of nanocomposites’ discoloration may be the absorbability of the material. Along with the absorption of water, microorganisms penetrate the interior of the polymer, as do dyes from the environment [48]. The evident tendency of significant discoloration, independent of the weight content of ZnO nanoparticles in PMMA in the case of red wine, cannot be explained solely by the content of ethanol. Alcohols, but also water as solvents, tend to penetrate the polymer mesh and can chemically soften polymeric dental materials. Water, as a complex solvent, because of its possible strong interaction with the polymer, due to its polarity and ability to form hydrogen bonds, has a tendency to cluster and cause plasticization of the material matrix. Ethanol enhances the plasticization, dissolution, and causes irreversible dental composite degradation by penetrating the matrix and expanding the space between polymer chains. The greatest discoloration in the case of red wine may be caused by the penetration of strong dyes in the form of polyphenols, and more precisely anthocyanins, into the spaces in the polymer mesh created by the ethanol solvent. In addition, the deposition of these highly colored substances can also take place mainly in the outer layer of the material due to the long-lasting tendency of ethanol to dissolve the unbound resin surface [49,50,51]. In the previous studies of the authors, it was proved that the absorbability of nanocomposites is up to 2%, which corresponds to the requirements of ISO standards (20795-1.2013 Dentistry-Base polymers-Part 1, Denture base polymers) for dentures. Small fluctuations in water absorption for individual nanocomposites do not explain the increased color change for the 7.5% nanocomposite.

The tendency of discoloration may also result from the very nature of the zinc oxide, the particles of which, when incorporated in the matrix, can change color. This phenomenon is often observed in clinical settings, where the ZnO-based temporary fillings left on for a longer period turn brown or become discolored upon contact with the oral environment. 

To overcome the limitations of the present study, it would be useful in future studies to evaluate a dynamic model that uses an experimental temperature range similar to human intraoral conditions. In our study, a static laboratory model was chosen, which seems to be one of the main limitations of this study. The colorants we chose are also consumed at different temperatures (hot or cold drinks, denture cleaners), so the model of subsequent studies should be supplemented with fluctuations associated with changes in temperature during the possible supply or use of these agents. The second limitation was the arbitrary imposition of the duration of the action of the coloring agents—the difficulties described above in the form of the lack of unambiguous models for recalculating the duration of the agents’ action in the oral cavity during the use of acrylic dentures require that the results of this study be perceived in the form of a material tendency rather than a clear effect during in vivo use. The third limitation—and at the same time, a direction for further studies—is the lack of microscopic or spectroscopic studies to assess the actual possibility of coloring agents interfering with the structure of polymethyl methacrylate, particularly with the admixture of nanoparticles in the form of a ZnO NP–PMMA nanocomposite.

## 5. Conclusions

Modification of PMMA with ZnO nanoparticles is aesthetically acceptable. However, slight whitening of the material (especially for 2.5% and 5% nanocomposites) must be discussed with the patient before potential clinical application. The use of a 7.5% nanocomposite is not recommended in clinical practice due to the high whitening of the material worsening the biomimetic properties and due to poor color stability that weakens the aesthetic effect.

## Figures and Tables

**Table 1:** Mean ± standard deviation of CIE L*a*b* values, ∆E in relation to control group, NBS, and statistical data of PMMA samples with different ZnO NP content.

CIE L*a*b* Coordinates		Group A PMMA (Control Group)	Group B PMMA-ZnONPs-2.5%	Group C PMMA-ZnONPs-5%	Group D PMMA-ZnONPs-7.5%	*p*
L*-Value	Mean ± SD	47.41 ± 1.15 ^1^	53.51 ± 0.32 ^1^	57.89 ± 1.67 ^1^	61.86 ± 1.09 ^1^	<0.000001 ^A^
	Min/Max	45.63/49.48	52.81/54.11	57.42/59.7	59.99/63.8	
a*-Value	Mean ± SD Min/Max	12.67 ± 0.66 ^1^ 11.48/14.15	11.64 ± 0.23 ^1^ 11.13/12.22	10.92 ± 0.41 ^2^ 10.36/11.88	10.12 ± 0.73 ^2^ 8.66/11.36	<0.000001 ^A^
b*-Value	Mean ± SD Min/Max	10.62 ± 0.78 ^1^ 8.93/11.71	6.02 ± 0.35 ^1^ 5.45/6.68	4.77 ± 0.70 ^3^ 3.72/6.47	5.01 ± 0.55 ^3^ 4.1/6.11	<0.000001 ^A^
Digital representation of mean color		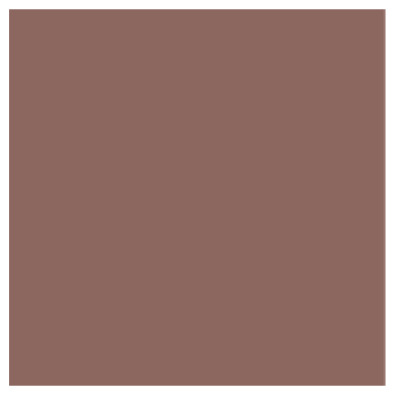	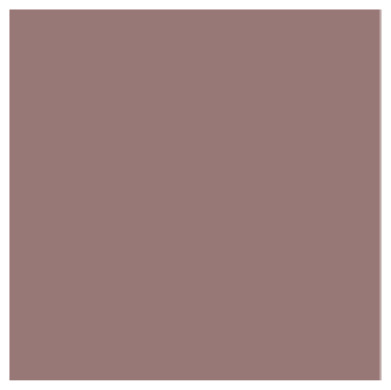	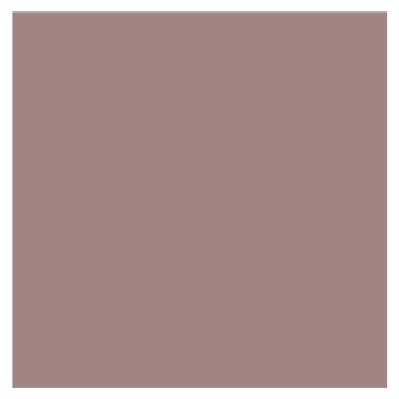	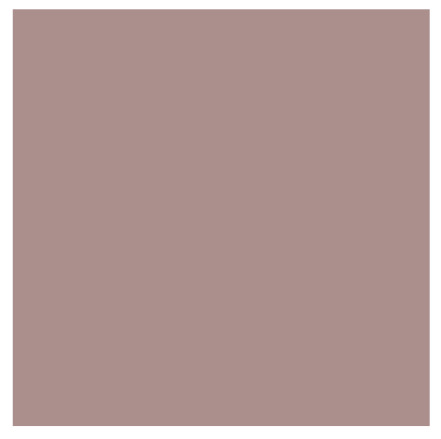	
∆Ε		0.000	7.705	12.128	15.708	
NBS		0.000	7.088	11.157	14.451	

^A^ statistically significance differences in ANOVA, ^1^ statistically significance in post hoc Tamhane’s with *p* < 0.000001, ^2^ statistically significance in post hoc Tamhane’s with *p* = 0.003285, ^3^ statistically significance in post hoc Tamhane’s with *p* = 0.00485.

## Data Availability

The data are not publicly available.

## References

[1] Almufleh B., Emami E., Alesawy A., Rodan R., Morris M., Umebayashi M., Tamimi F. (2020). Patient-Reported Outcomes of Metal and Acrylic Resin Removable Partial Dentures: A Systematic Review and Meta-Analysis. J. Prosthodont..

[2] Zafar M.S. (2020). Prosthodontic Applications of Polymethyl Methacrylate (PMMA): An Update. Polymers.

[3] Kumar M.V., Bhagath S., Jei J.B. (2010). Historical interest of denture base materials. J. Dent. Sci..

[4] Ali Sabri B., Satgunam M., Abreeza N.M., Abed A.N., Jones I.P. (2021). A review on enhancements of PMMA Denture Base Material with Different Nano-Fillers. Cogent Eng..

[5] Ratanajanchai M., Kanchanavasita W., Suputtamongkol K., Wonglamsam A., Thamapipol S., Sae-Khow O. (2021). Heat-cured poly(methyl methacrylate) resin incorporated with different food preservatives as an anti-microbial denture base material. J. Dent. Sci..

[6] Rashin G., Firouzmandi M., Neda Zare K., Ansarifard E. (2022). Influence of different concentrations of titanium dioxide and copper oxide nanoparticles on water sorption and solubility of heat-cured PMMA denture base resin. Clin. Exp. Dent. Res..

[7] Murakami N., Wakabayashi N., Matsushima R., Kishida A., Igarashi Y. (2013). Effect of high-pressure polymerization on mechanical properties of PMMA denture base resin. J. Mech. Behav. Biomed. Mater..

[8] Cierech M., Szczypińska A., Wróbel K., Gołaś M., Walke W., Pochrząst M., Przybyłowska D., Bielas W. (2013). Comparative analysis of the roughness of acrylic resin materials used in the process of making the bases of dentures and investigation of adhesion of Candida albicans to them. In vitro study. Dent. Med. Probl..

[9] Sushma R., Vande A.V., Malvika S.R., Abhijeet K., Pronob K.S. (2018). A comparative study of the mechanical properties of clear and pink colored denture base acrylic resins. Ann. Afr. Med..

[10] Gad M.M., Fouda S.M., Al-Harbi F.A., Näpänkangas R., Aune R. (2017). PMMA denture base material enhancement: A review of fiber, filler, and nanofiller addition. Int. J. Nanomed..

[11] Scarano A., Orsini T., Di Carlo F., Valbonetti L., Lorusso F. (2021). Graphene-Doped Poly (Methyl-Methacrylate) (Pmma) Implants: A Micro-CT and Histomorphometrical Study in Rabbits. Int. J. Mol. Sci..

[12] Awad M.A., Hendi A.A., Ortashi K.M.O., Alanazi A.B., Alzahrani B.A., Soliman D.A. (2019). Greener Synthesis, Characterization, and Antimicrobiological Effects of *Helba* Silver Nanoparticle-PMMA Nanocomposite. Int. J. Polym. Sci..

[13] Chladek G., Mertas A., Barszczewska-Rybarek I., Nalewajek T., Żmudzki J., Król W., Łukaszczyk J. (2011). Antifungal Activity of Denture Soft Lining Material Modified by Silver Nanoparticles—A Pilot Study. Int. J. Mol. Sci..

[14] Nam K.-Y., Lee C.-H., Lee C.-J. (2012). Antifungal and physical characteristics of modified denture base acrylic incorporated with silver nanoparticles. Gerodontology.

[15] Sun J., Wang L., Wang J., Li Y., Zhou X., Guo X., Zhang T., Guo H. (2021). Characterization and evaluation of a novel silver nanoparticles-loaded polymethyl methacrylate denture base: In vitro and in vivo animal study. Dent. Mater. J..

[16] Sokołowski K., Szynkowska M.I., Pawlaczyk A., Łukomska-Szymańska M., Sokołowski J. (2014). The impact of nanosilver addition on element ions release form light-cured dental composite and compomer into 0.9% NaCl. Acta Biochim. Pol..

[17] Cierech M., Wojnarowicz J., Kolenda A., Łojkowski W., Mierzwińska-Nastalska E., Zawadzki P. (2017). Characteristics of titanium nano-oxide (IV) as potent polymethyl metacrylate modifier. Prosthodontics.

[18] Cierech M., Szerszeń M., Wojnarowicz J., Łojkowski W., Kostrzewa-Janicka J., Mierzwińska-Nastalska E. (2020). Preparation and characterization of poly(Methyl metacrylate)-titanium dioxide nanocomposites for denture bases. Polymers.

[19] Liu X., Wang Z., Zhao C., Bu W., Na H. (2018). Preparation and characterization of silane-modified SiO2 particles reinforced resin composites with fluorinated acrylate polymer. J. Mech. Behav. Biomed. Mater..

[20] Oral O., Lassila L.V., Kumbuloglu O., Vallittu P.K. (2014). Bioactive glass particulate filler composite: Effect of coupling of fillers and filler loading on some physical properties. Dent. Mater..

[21] Silva T.M.D., Sales A., Pucci C.R., Borges A.B., Torres C.R.G. (2017). The combined effect of food-simulating solutions, brushing and staining on color stability of composite resins. Acta Biomater. Odontol. Scand..

[22] Özdaş D., Kazak M., Çilingir A., Subaşı M.G., Tiryaki M., Günal Ş. (2016). Color Stability of Composites after Short-term Oral Simulation: An in vitro Study. Open Dent. J..

[23] Paolone G., Formiga S., De Palma F., Abbruzzese L., Chirico L., Scolavino S., Goracci C., Cantatore G., Vichi A. (2022). Color stability of resin-based composites: Staining procedures with liquids-A narrative review. J. Esthet. Restor. Dent..

[24] Cierech M., Kolenda A., Grudniak A.M., Wojnarowicz J., Woźniak B., Gołaś M., Swoboda-Kopeć E., Łojkowski W., Mierzwińska-Nastalska E. (2016). Significance of polymethylmethacrylate (PMMA) modification by zinc oxide nanoparticles for fungal biofilm formation. Int. J. Pharm..

[25] Cierech M., Wojnarowicz J., Kolenda A., Krawczyk-Balska A., Prochwicz E., Woźniak B., Łojkowski W., Mierzwińska-Nastalska E. (2019). Zinc oxide nanoparticles cytotoxicity and release from newly formed PMMA–ZnO nanocomposites designed for denture bases. Nanomaterials.

[26] Lopes-Rocha L., Mendes J.M., Garcez J., Sá A.G., Pinho T., Souza J.C.M., Torres O. (2021). The Effect of Different Dietary and Therapeutic Solutions on the Color Stability of Resin-Matrix Composites Used in Dentistry: An In Vitro Study. Materials.

[27] Witzel R.F., Burnham R.W., Onley J.W. (1973). Threshold and suprathreshold perceptual color differences. J. Opt. Soc. Am..

[28] Mokrzycki W., Tatol M. (2011). Color difference Delta–E—A survey. Mach. Graph. Vis..

[29] Cal E., Güneri P., Kose T. (2006). Comparison of digital and spectrophotometric measurements of color shade guides. J. Oral Rehabil..

[30] Chu S., Trushkowsky R., Paravina R. (2010). Dental color matching instruments and systems. Review of clinical and research aspects. J. Dent..

[31] Paravina R.D., Pérez M.M., Ghinea R. (2019). Acceptability and perceptibility thresholds in dentistry: A comprehensive review of clinical and research applications. J. Esthet. Restor. Dent..

[32] Wojnarowicz J., Chudoba T., Koltsov I., Gierlotka S., Dworakowska S., Lojkowski W. (2018). Size control mechanism of ZnO nanoparticles obtained in microwave solvothermal synthesis. Nanotechnology.

[33] Wojnarowicz J., Chudoba T., Lojkowski W. (2020). A Review of Microwave Synthesis of Zinc Oxide Nanomaterials: Reactants, Process Parameters and Morphologies. Nanomaterials.

[34] Majcher A., Wiejak J., Przybylski J., Chudoba T., Wojnarowicz J. (2013). A Novel Reactor for Microwave Hydrothermal Scale-up Nanopowder Synthesis. Int. J. Chem. React. Eng..

[35] Polish Center for Accreditation, Testing Laboratories. Accreditation Number: AB 1503. https://www.pca.gov.pl/en/accredited-organizations/accredited-organizations/testing-laboratories/AB%201503,entity.html.

[36] Cierech M., Wojnarowicz J., Szmigiel D., Bączkowski B., Grudniak A., Wolska K., Łojkowski W., Mierzwińska-Nastalska E. (2016). Preparation and characterization of ZnO-PMMA resin nanocomposites for denture bases. Acta Bioeng. Biomech..

[37] Rudolf R., Popović D., Tomić S., Bobovnik R., Lazić V., Majerič P., Anžel I., Čolić M. (2020). Microstructure Characterisation and Identification of the Mechanical and Functional Properties of a New PMMA-ZnO Composite. Materials.

[38] Cierech M., Szerszeń M., Wojnarowicz J., Łojkowski W., Kostrzewa-Janicka J., Mierzwińska-Nastalska E. (2020). Colorimetric study of zinc oxide poly(methyl methacrylate) nanocomposite—New biomaterial for denture bases. Prosthodontics.

[39] Kamonkhantikul K., Arksornnukit M., Takahashi H. (2017). Antifungal, optical, and mechanical properties of polymethylmethacrylate material incorporated with silanized zinc oxide nanoparticles. Int. J. Nanomed..

[40] Elagra M.I., Rayyan M.R., Alhomaidhi M.M., Alanaziy A.A., Alnefaie M.O. (2017). Color stability and marginal integrity of interim crowns: An in vitro study. Eur. J. Dent..

[41] Alhotan A., Elraggal A., Yates J., Haider J., Jurado C.A., Silikas N. (2022). Effect of Different Solutions on the Colour Stability of Nanoparticles or Fibre Reinforced PMMA. Polymers.

[42] Babikir M.O., Gilada M.W., Fahmy F., Ismail I.A., Alhajj M.N., Fadul A.A., Elasyouti A. (2019). Effect of Commonly Consumed Beverages on Color Stability of Polymethyl Methacrylate Denture Base Material. Compend. Contin. Educ. Dent..

[43] Dimitrova M., Corsalini M., Kazakova R., Vlahova A., Barile G., Dell’Olio F., Tomova Z., Kazakov S., Capodiferro S. (2022). Color Stability Determination of CAD/CAM Milled and 3D Printed Acrylic Resins for Denture Bases: A Narrative Review. J. Compos. Sci..

[44] Gad M.M., Abualsaud R., Fouda S.M., Rahoma A., Al-Thobity A.M., Khan S.Q., Akhtar S., Al-Abidi K.S., Ali M.S., Al-Harbi F.A. (2021). Color Stability and Surface Properties of PMMA/ZrO_2_ Nanocomposite Denture Base Material after Using Denture Cleanser. Int. J. Biomater..

[45] De Kok I.J., Cooper L.F., Guckes A.D., McGraw K., Wright R.F., Barrero C.J., Bak S.Y., Stoner L.O. (2017). Factors Influencing Removable Partial Denture Patient-Reported Outcomes of Quality of Life and Satisfaction: A Systematic Review. J. Prosthodont..

[46] Heimer S., Schmidlin P.R., Stawarczyk B. (2017). Discoloration of PMMA, composite, and PEEK. Clin. Oral Investig..

[47] Taylor M., Masood M., Mnatzaganian G. (2022). Differences in complete denture longevity and replacement in public and private dental services: A propensity score-matched analysis of subsidised dentures in adult Australians across 20 years. Community Dent. Oral Epidemiol..

[48] Kucharski Z. (2008). Physical properties of resilient materials in prosthodontics. Prosthodontics.

[49] Polydorou O., Trittler R., Hellwig E., Kümmerer K. (2007). Elution of monomers from two conventional dental composite materials. Dent. Mater..

[50] Regis R.R., Soriani N.C., Azevedo A.M., Silva-Lovato C.H., Paranhos H.F., de Souza R.F. (2009). Effects of ethanol on the surface and bulk properties of a microwave-processed PMMA denture base resin. J. Prosthodont..

[51] Basavarajappa S., Al-Kheraif A.A.A., ElSharawy M., Vallittu P.K. (2016). Effect of solvent/disinfectant ethanol on the micro-surface structure and properties of multiphase denture base polymers. J. Mech. Behav. Biomed. Mater..

